# Results from a community-based program evaluating the effect of changing smoking status on asthma symptom control

**DOI:** 10.1186/1471-2458-12-293

**Published:** 2012-04-20

**Authors:** Teresa To, Corinne Daly, Rachel Feldman, Susan McLimont

**Affiliations:** 1 Child Health Evaluative Sciences, The Hospital for Sick Children, 555 University Avenue, Toronto, Ontario, M5G 1X8, Canada; 2 Institute for Clinical Evaluative Sciences, G1 06-2075 Bayview Avenue, Toronto, Ontario, M4N 3M5, Canada; 3 Dalla Lana School of Public Health, University of Toronto, 155 College Street, Toronto, Ontario, M5T 3M7, Canada; 4 Institute of Medical Science, University of Toronto, 1 King’s College Circle, Toronto, Ontario, M5S 1A8, Canada

## Abstract

**Background:**

Cigarette smoking has been associated with accelerated decline in lung function, increased health services use and asthma severity in patients with asthma. Previous studies have provided insight into how smoking cessation improves lung function among asthma patients, however, fail to provide measurable asthma symptom-specific outcomes after smoking cessation. The objective of this study was to measure the effect of changing smoking status on asthma symptom control and health services use in adults with asthma.

**Methods:**

The study was conducted in eight primary care practices across Ontario, Canada participating in a community-based, participatory, and evidence-based Asthma Care Program. Patients aged 18 to 55 identified with physician-diagnosed mild to moderate asthma were recruited. In addition to receiving clinical asthma care, participants were administered a questionnaire at baseline and 12-month follow-up visits to collect information on demographics, smoking status, asthma symptoms and routine health services use. The effect of changing smoking status on asthma symptom control was compared between smoking groups using Chi-square and Fisher’s exact tests where appropriate. Mixed effect models were used to measure the impact of the change in smoking status on asthma symptom and health services use while adjusting for covariates.

**Results:**

This study included 519 patients with asthma; 11% of baseline smokers quit smoking while 4% of baseline non-smokers started smoking by follow-up. Individuals who quit smoking had 80% lower odds of having tightness in the chest (Odds ratio (OR) = 0.21, 95% CI: 0.06, 0.82) and 76% lower odds of night-time symptoms (OR = 0.24, 95% CI: 0.07, 0.85) compared to smokers who continued to smoke. Compared to those who remained non-smokers, those who had not been smoking at baseline but self-reported as current smoker at follow-up had significantly higher odds of chest tightness (OR = 1.36, 95% CI: 1.10, 1.70), night-time symptoms (OR = 1.55, 95% CI: 1.09, 2.20), having an asthma attack in the last six months (OR = 1.43, 95% CI: 1.17, 1.75) and visiting a walk-in clinic for asthma (OR = 4.57, 95% CI: 1.44, 14.49).

**Conclusions:**

This study provides practitioners measurable and clinically important findings that associate smoking cessation with improved asthma control. Health practitioners and asthma programs can use powerful education messages to emphasize the benefits of smoking cessation as a priority to current smokers.

## Background

Smoking has been associated with accelerated decline in lung function, increased health services use and asthma severity in patients with asthma [1]. For these patients that smoke, they experience worse control over their asthma as compared with non-smokers with asthma [2]. Previously reported questionnaire and telephone surveys of individuals with asthma provide conclusive evidence that current smoking among asthma patients is associated with a failure to obtain suitable levels of asthma control [1-3]. In turn, smoking cessation among asthma patients has led to improved lung function, reduced use of β_2_-agonists, lower doses of inhaled corticosteroids required, less frequent daytime asthma symptoms and higher asthma-specific quality of life scores [4].

One study investigating the short-term effect of smoking cessation on lung function, airway inflammation and responsiveness in 20 smokers with asthma found that those who quit smoking had achieved improvements in lung function and reduction in sputum neutrophil by 6 weeks after they stopped smoking [5]. In a larger scale study, involving 3,197 asthma patients, the effects of inhaled corticosteroids on asthma symptom control were lessened by cigarette smoking; highlighting the need to place more emphasis on smoking cessation as part of asthma education for patients to ensure treatment success [6].

While these studies have provided insight into how smoking cessation improves lung function among asthma patients they fall short in providing asthma symptom-specific outcomes after smoking cessation. Additionally, there still remains little documented literature on the effect of smoking cessation on asthma symptom control across a longitudinal period and among smokers. The aim of this study was to measure the effect of changing smoking status on asthma symptom control and health services use in adults with asthma who participated in a comprehensive evidence-based asthma program in order to provide health care providers meaningful information pertinent to educating patients with asthma who smoke.

## Methods

### Study setting

The Primary Care Asthma Pilot Project (PCAPP) was conducted between January 2003 and May 2006 in the province of Ontario, Canada, and has been previously described [7,8]. In brief, the study was conducted in eight primary care practices comprising 15 satellite clinics across the largest province in Canada. These sites resided in inner-city, urban and rural communities as well as one isolated Northern Aboriginal community and provide patient care through multidisciplinary health care teams (including general family practitioners, nurses and nurse practitioners, medical residents and social workers). The study methodology and materials were reviewed and approved by the Research Ethics Board at The Hospital for Sick Children Research Institute, Toronto, Ontario.

### Study design and procedure

The study was a community-based participatory pre and post design to evaluate implementation of the evidence-based Asthma Care Program. This program is based on asthma management standards developed by the Canadian Thoracic Society Canadian Asthma Consensus Guidelines [9-13] and consists of five components: 1) an asthma care map; 2) a treatment flow chart; 3) program standards; 4) a written asthma action plan; and 5) core elements of asthma education. Prior to implementation, the asthma care map was developed by the Ontario Thoracic Society, as part of the Ontario Ministry of Health and Long-Term Care’s Asthma Plan of Action, for use by a multi-disciplinary team of primary health care providers as a template for guideline-based management. It incorporates all elements of the Canadian Asthma Consensus Guidelines, including assessment and diagnosis, drug therapy and treatment plan, action plan, patient education, and environmental control.

A designated study coordinator (Certified Asthma Educators, respiratory therapists or nurses with experience in asthma education) was assigned to each site and was responsible for implementation of the Asthma Care Program, performing spirometry, providing asthma education to participants, coordinating program activities, and recruiting participants. The study coordinators approached patients aged 2 to 55 that were identified as having been diagnosed with asthma by a physician to participate in the study and then obtained consent. Patients with unclear diagnosis of asthma, physician-diagnosed Chronic Obstructive Pulmonary Disease (COPD) or any of foreign body airway obstruction, congenital heart disease, bronchopulmonary dysplasia, Alzheimer’s disease, dementia, alcoholism or significant neurological deficit were excluded.

In addition to the provision of clinical asthma care at participants’ baseline and follow-up visit at 12-months, a questionnaire was administered by the site study coordinator at both time points to collect demographic information, smoking status, asthma symptoms, acute and routine health services use, asthma management, and medication use.

### Independent variables

At baseline, demographic information was collected from patients using the questionnaire and included date of birth, gender, level of education, household income, household size, and whether participants had a drug benefit plan to pay for the cost of their asthma medications. Using household income and family size, a proxy measure of socioeconomic status (SES) was derived. A family of four or more with an annual income of less than $40,000 (Canadian dollars) was defined as low SES based on the 2002 before tax Low Income Cut-offs as calculated by Statistics Canada [14].

Smoking status was defined by the participant response to the following question that was asked to at the baseline and 12-month follow-up visit, “A*t the present time, do you smoke cigarettes*?” The four groups of smoking status were: 1) *smokers* (those who were current smokers at both baseline and follow-up), 2) *ex-smokers* (those who were smokers at baseline but not at follow-up), 3) *new smokers* (those who were non-smokers at baseline but were current smokers at follow-up), and 4) *non-smokers * (those who were non-smokers at both baseline and follow-up). Frequency of smoking (i.e. daily, weekly or less than a week) was also collected, however, for the purpose of this study any participant answering yes to the smoking status question was considered to be smoking at that time irrespective of the frequency reported.

### Outcome measures

At baseline and 12-month follow-up, self-reported data on asthma symptom control and health services use were collected. Asthma control was defined as the presence of wheezing, shortness of breath, chest tightness and/or cough, in the previous four weeks, daytime asthma symptoms more than three times per week or night-time symptoms one or more times per week, at least one asthma exacerbation in the previous six months, and use of asthma preventer medication in the previous six months. Asthma health services use was defined as at least one emergency department visit in the previous six months and at least one urgent care visit (physician, walk-in clinic or urgent care centre) in the previous six months.

### Statistical analysis

Only those aged 18 years and older (adults) were included in this study for analysis. Baseline characteristics including demographics, health status, and asthma control of the four smoking status groups were compared using Chi-square and Fisher’s exact tests where appropriate. The statistical significance in the change of asthma outcome measures (symptoms and health services use) between baseline and 12-month follow-up was measured by the McNemar’s test. Mixed effect models were used to measure the impact of the change in smoking status on asthma symptom and health services use while adjusting for other covariates. Each outcome measure at 12-month follow-up (listed above) was modelled against its respective baseline measure and adjusted for age and sex. The mixed effect models were repeated comparing smokers to ex-smokers and non-smokers to new smokers separately. Analyses were performed using SAS version 9.1 (SAS Institute Inc., Cary, NC).

## Results

### Study population baseline characteristics by smoking status

**Figure 1:**
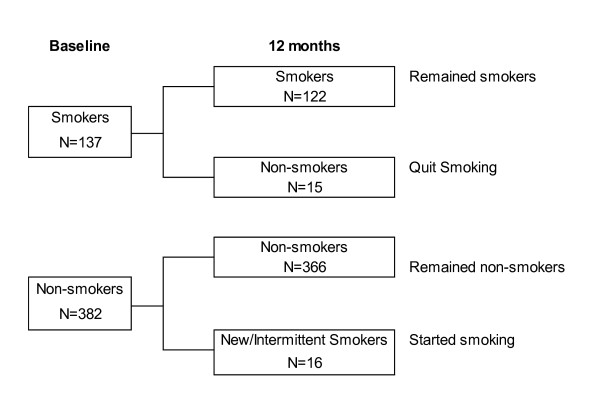
Number of individuals participating in study categorized according to smoking status at baseline and follow-up.

A total of 733 participants aged 18 to 55 years were recruited at baseline, and of those 519 (70.8%) who had information on smoking status at both baseline and 12-month follow-up. Figure 1 showed that of the 519 participants included in this study, 137 (26.4%) were smokers at baseline. Amongst the baseline smokers, most (n = 122, 89.1%) remained smokers, while 15 (10.9%) quit smoking at the 12-month follow-up visit. Of the 382 baseline non-smokers, the majority of them (n = 366, 95.8%) remained non-smokers, while only a small proportion (n = 16, 4.2%) started smoking by the 12-month follow-up visit. Among the baseline smokers, those who remained smokers and those who quit smoking at 12-months were not statistically different by demographic characteristics (Table 1). Baseline non-smokers who became smokers at 12-months were younger (35.3 ± 6.9 versus 40.8 ± 9.8, p = 0.007) and a higher proportion with a drug plan (56.3% versus 22.9%, p = 0.005) compared to subjects who remained non-smokers.

**Table 1:** Demographic characteristics and smoking frequency at baseline/follow-up by smoking groups

	**Smokers**		Ex-smokers*			**Non-smokers**		New Smokers*		
	**n**	**%**	**n**	**%**	**p-value**	**n**	**%**	**n**	**%**	**p-value**
**Number of Patients**	122		15			366		16		
**Baseline demographics**										
Age (Mean ± SD)	39.13 ± 9.33		42.72 ± 8.10		0.128	40.82 ± 9.79		35.25 ± 6.93		0.007
Females	99	81.1	12	80.0	1.000	285	77.9	11	68.8	0.370
Residence					0.321					0.753
Urban Area	12	9.8	3	20.0		91	24.9	3	18.8	
Rural Area	48	39.3	7	46.7		146	39.9	6	37.5	
Inner City Area	62	50.8	5	33.3		129	35.2	7	43.8	
Education (university/college or above)	44	36.1	5	33.3	0.818	248	67.8	10	62.5	0.660
Family Income ≤ $40,000	13	10.7	0	0.0	0.360	36	9.8	2	12.5	0.667
Without a Drug Plan	36	29.5	4	26.7	0.942	83	22.9	9	56.3	0.005
**Smoking Frequency at Baseline**										
Daily	113	92.6	10			n/a		n/a		
Weekly	2	1.6	1			n/a		n/a		
Less than once per week	7	5.7	4			n/a		n/a		
**Smoking Frequency at 12-month Follow-up†**										
Daily	110	90.2	n/a			n/a		11		
Weekly	4	3.3	n/a			n/a		2		
Less than once per week	6	4.9	n/a			n/a		3		

*denotes smoking status at 12-month follow-up.

†missing data for 2 smokers at follow-up.

Abbreviations: SD, standard deviation; n/a, not applicable.

### Asthma symptoms at baseline and 12-month follow-up

Table 2 showed the percent distributions of asthma symptoms and health services use at baseline and 12-month follow-up by smoking status. Overall, the ex-smokers showed the biggest improvement in asthma symptom control over time and had the lowest level of self-reported wheeze, shortness of breath, tightness of chest and day-time symptoms amongst the four groups. Additionally, these individuals showed a significant decrease in shortness of breath, chest tightness and night-time symptoms compared to their smoking counterparts at follow-up.

**Table 2:** Percent distributions of asthma symptoms and health services use at baseline and 12-month follow-up by smoking status

	**Baseline**						**12-Month Follow-up**					
	**Smokers**	**Ex-Smokers**	**p-value**	**Non-Smokers**	**New Smokers**	**p-value**	**Smokers**	**Ex-Smokers**	**p-value**	**Non-Smokers**	**New Smokers**	**p-value**
Number of Patients	122	15		366	16		122	15		366	16	
**Health Status**												
Fair-Poor Health rating	43.7	28.6		72.7	53.3		35.5	13.3		16.4	31.3	
Less active	45.1	33.3		62.3	56.3		42.6	33.3		25.3	31.3	
**Asthma Control**												
*Symptoms (last 4 weeks)*												
Wheeze	80.3	80.0		63.8	87.5		66.4	33.3	*	48.9	81.3	*
Shortness of Breath	82.0	66.7		76.8	93.8		63.9	53.3		60.4	75.0	
Chest Tightness	77.1	53.3		68.0	81.3		60.7	13.3	**	50.3	62.5	
Cough	85.3	73.3		68.6	75.0		78.7	66.7		56.9	62.5	
Night-time Symptoms	72.7	66.7		61.7	75.0		54.1	13.3	*	40.2	68.8	*
Day-time Symptoms	96.7	93.3		90.7	93.8		90.2	80.0		80.3	100.0	
> 1 Asthma Attack in Last 6 months	81.7	61.5		79.3	76.9		63.9	33.3		56.6	60.0	
*Acute Health Services Use (last 6 months)*												
Any Urgent Visit	12.3	33.3	*	18.0	18.8		16.4	13.3		13.1	18.8	
ER Visits	1.6	6.7		8.2	18.8		8.2	6.7		6.8	0.0	
Walk-in Clinic Visits	11.5	26.7		12.9	0.0		8.2	13.3		7.1	18.8	
*School/Work Absenteeism (last 4 weeks)*												
Missed Work/School	18.5	30.8		8.6	40.0	*	10.7	0.0		10.1	13.3	
**Asthma Management**												
Received asthma education	43.4	33.3		39.9	43.8		24.8	13.3		23.2	62.5	*
Given an action plan	9.5	6.7		19.8	25.0		34.2	40.0		31.5	37.5	
Regular peakflow monitoring	16.5	40.0	*	24.5	25.0		40.3	40.0		49.5	43.8	
Spirometry	76.3	78.6		69.3	60.0		93.3	100.0		96.4	87.5	
**Medication Use**												
Preventer use	73.8	86.7		71.0	81.3		79.5	73.3		73.5	75.0	
Reliever use	82.0	86.7		86.6	87.5		85.3	80.0		83.3	93.8	

*p<0.05, ** p<0.001.

At 12-month follow-up, in general, the prevalence of asthma symptoms (in last 4 weeks) lessened from baseline in all four groups, except for the new smokers who experienced slightly more day-time symptoms. At 12-month follow-up, the level of wheeze (33.3% versus 66.4%, p = 0.012), tightness in the chest (13.3% versus 60.7%, p = 0.001), and night-time symptoms (13.3% versus 54.1%, p = 0.003) in the ex-smokers were significantly lower when compared to those who remained smokers. In contrast, the levels of wheeze (81.3% versus 48.9%, p = 0.011) and night-time symptoms (68.8% versus 40.2%, p = 0.023) among the new smokers were significantly higher when compared to those who remained non-smokers.

The percentage of individuals who had more than one asthma attack in the last 6 months also decreased in all four groups, with the ex-smokers showing almost 50% decrease in 12 months.

### Asthma health services use at baseline and 12-month follow-up

At baseline and among the smokers, those who remained smokers and those who quit smoking at 12-month follow-up were not statistically different except that ex-smokers had almost 3 times higher urgent visits for asthma (33.3% versus 12.3%, p = 0.045) and higher proportion of them had peak flow monitoring (40.0% versus 16.5%, p = 0.04). Among the non-smokers, the new smokers had a higher percentage of school/work absenteeism at baseline (40.0% versus 8.6%, p = 0.002).

The use of health services for asthma in the last 6 months was reported by the participants. While all other groups had either no change or a decrease in emergency department visits, the smokers had a five-fold increase from baseline (from 1.6% to 8.2%, p = 0.0209). The ex-smokers had the largest decrease in asthma health services use, accompanied by a decrease in the use of preventer and reliever medications; however, these differences were not statistically significant.

### Mixed effect models

Table 3 showed the impact of the change in smoking status on asthma symptom and health services use analysed using mixed effect models while adjusting for age, sex, baseline symptoms and health services use. Individuals who quit smoking had 80% lower odds of having tightness in the chest (OR = 0.21, 95% CI: 0.06, 0.82) and 76% lower odds of night-time symptoms (OR = 0.24, 95% CI: 0.07, 0.85) compared to smokers who continued to smoke. On the other hand, compared to the non-smokers, new smokers had significantly higher odds of tightness in the chest (OR = 1.36, 95% CI: 1.10, 1.70), night-time symptoms (OR = 1.55, 95% CI: 1.09, 2.20), having an asthma attack in the last 6 months (OR = 1.43, 95% CI: 1.17, 1.75) and encountering a visit to the walk-in clinic for asthma (OR = 4.57, 95% CI: 1.44, 14.49).

**Table 3:** Mixed effect models to model impact of change in smoking status on asthma symptoms and health services use

	**Ex-Smokers versus Smokers (reference)**	**New/Intermittent Smokers versus Non-Smokers (reference)**
**Outcome Measures**	**Odds Ratio 95% CI**	**Odds Ratio 95% CI**
**Asthma Symptoms**		
*Wheeze*		
Crude	0.50[0.24,1.04]	1.66[1.28,2.15]**
Adjusted^1^	0.51[0.25,1.02]	1.32[0.98,1.76]
*Shortness of Breath*		
Crude	0.83[0.51,1.36]	1.24[0.92,1.67]
Adjusted	0.85[0.52,1.37]	1.07[0.79,1.45]
*Chest Tightness*		
Crude	0.22[0.06,0.81]*	1.24[0.84,1.84]
Adjusted	0.21[0.06,0.82]*	1.36[1.10,1.70]*
*Cough*		
Crude	0.85[0.59,1.23]	1.10[0.74,1.62]
Adjusted	0.87[0.63,1.21]	1.03[0.69,1.52]
*Night-time Symptoms*		
Crude	0.25[0.07,0.90]*	1.71[1.20,2.44]*
Adjusted	0.24[0.07,0.85]*	1.55[1.09,2.20]*
*Day-time Symptoms*		
Crude	0.89[0.68,1.15]	NA
Adjusted	0.94[0.78,1.14]	NA
*> 1 Asthma Attack in last 6 months*		
Crude	0.52[0.23,1.18]	1.06[0.69,1.62]
Adjusted	0.56[0.22,1.39]	1.43[1.17,1.75]**
**Acute Health Services Use (last 6 months)**		
*Any Urgent Visit*		
Crude	0.81[0.21,3.14]	1.43[0.50,4.10]
Adjusted	0.78[0.21,2.94]	1.31[0.39,4.39]
*ER Visits*		
Crude	0.81[0.11,5.92]	NA
Adjusted	NA	NA
*Walk-in Clinic Visits*		
Crude	1.63[0.39,6.73]	2.64[0.89,7.81]
Adjusted	NA	4.57[1.44,14.49]*
**School/Work Absenteeism (last 4 weeks)**		
*Missed Work/School*		
Crude	NA	1.32[0.35,4.99]
Adjusted	NA	1.30[0.30,5.64]

^1^Multivariable mixed effect models adjusted for age, sex, baseline symptoms and health services use.* p<0.05, ** p<0.001.

NA, not available due to small sample size.

## Discussion

This community-based participatory study evaluating the effect of a change in smoking status on asthma symptom control and health services use among adults living with asthma over a 12 month period shows that asthma patients who quit smoking experience significantly lower (80%) night-time symptoms and chest tightness than individuals who continue to smoke. In contrast, non-smokers who took up smoking during follow-up had significantly higher risks of asthma symptoms, 1.4-fold higher risk of having asthma attacks and nearly 5-fold risk of having visits to walk-in clinics for asthma.

It is important for health care providers to encourage asthma patients who smoke to quit. The emphasis for health care providers to take an active role and use strategies to educate asthma patients about the benefits of smoking cessation is well documented [15-17]. Unfortunately, the success rates for smoking cessation in those with asthma appears to be disappointingly low and evidence on the effectiveness of smoking cessation in asthma is rather limited [4-6]. Although health care providers may have tools to educate their patients about the impact of quitting smoking, they may still be lacking points of reference and achievable benefits of behavioural change that can be discussed with patients. In order to positively impact efforts of smoking cessation, a system that supports both patients and their health care team is required to successfully change patient behaviour. According to the Centers for Disease Control in the US, [18] the majority of cigarette smokers quit without using evidence-based cessation treatments. However, there are other proven effective treatments: 1) brief clinical interventions (i.e. when a doctor takes 10 minnutes or less to deliver advice and assistance about quitting), 2) counselling, 3) behavioural cessation therapies, 4) treatments with more person-to-person contact and intensity, and 5) cessation medications found to be effective for treating tobacco dependence. To ensure the effectiveness of these approaches, such a system should ideally facilitate health care providers to make referrals and to be reimbursed accordingly for counselling. Furthermore, such a system would ensure patients while quitting smoking receive ongoing support in their routine asthma care follow-up from health care providers.

Some providers may inform smoking patients that they will experience improvement in lung function as early as one week following smoking cessation with a further improvement up to six weeks after quitting, as shown by Chaudhuri et al. [5]. However, patients may not associate lung function improvement with “feeling better” and having “well-controlled” asthma; instead, improvements in asthma control may be more sought after. While we did not have lung function data to indicate pulmonary improvement, our asthma symptom control data provides similar positive evidence that smoking cessation is significantly associated with lower asthma symptoms within 12 months following cessation.

Our study findings offer health care providers measurable clinical benefits that can be imparted upon asthma patients about the benefits of quitting smoking. An example of a patient education message may include, “*After you have stopped smoking for 12 months, the odds of feeling chest tightness is lowered by 80% and the risk of night-time symptoms by 75% compared to an individual who is still smoking.*” It is equally important to emphasize to current non-smokers that smoking is one of the significant risk factors for asthma morbidity. Asthma patients who are currently non-smokers should be encouraged to remain so by their health care providers. For example, communicate to non-smokers that, “*If you start smoking, your odds of wheezing will be increased by 30%, night-time symptoms by 50% and having an asthma attack (within a 6-month period) by 40%”.* Health care providers can convey the clinical benefits or risks associated with asthma and smoking to patients using such powerful and practical messages.

There is a large body of literature suggesting different methods to present treatment effects to patients that may affect health care decisions. Previously, it has been demonstrated that presenting benefits of specific treatments or behaviour to patients as a relative risk reduction with a comparator is a successful tactic [19]. Fagerlin et al [20] found that providing average risk information was valuable for physicians promoting patient behaviour with smoking cessation. Our suggested messages to health care providers agree with what was suggested in the literature about using relative risk reduction information and referencing it with a control comparator. Whether over time this tactic yields affective change will have to be demonstrated in a long-term follow-up study and is beyond the scope of this current study. Interestingly, we found that baseline non-smokers who were smoking at 12-month follow-up had an odds ratio of 4.57 of a walk-in clinic visit compared to patients that remained non-smokers. Previously, studies have found that smoking was associated with a greater longitudinal risk of hospitalization for asthma [21] and found that persons who visited an emergency room for asthma in the past 12 months were 60% more likely to be smokers [3].

While baseline smokers who quit by the 12-month follow-up visit experience improvements in asthma symptom control, individuals who continue to smoke might not show improvement due to the use of inhaled corticosteroids (ICS). Smokers with asthma compared with non-smokers with asthma are less sensitive to the short-term and medium-term effects of ICS on symptoms and lung function [22-24], suggesting that ICS doses may need to be adjusted to attain asthma control in smokers.

The approach used in our current study is novel. To the best of our knowledge, the only published Canadian study comparing adult asthma patients’ symptom control among smoking status groups was based on a telephone survey conducted by Boulet et al. [1]. The study assessed self-reported asthma control and health services use among individuals with asthma who were current smokers, had quit smoking or were never smokers. Patients with asthma who smoked were found to have worse control of their asthma than those who had stopped smoking or who had never smoked. However, this study was limited by the cross-sectional design and was unable to measure changes in health outcomes associated with the change in smoking status. In contrast, our study was a community-based face-to-face prospective study where asthma outcome measures were collected and assessed by health care practitioners (family doctors, nurses and asthma educators). The current study measured multiple outcomes including asthma medication use, work/school absenteeism, health status and asthma-related health services use. Most previous published studies have mainly focused on only one of these outcome measures [4,6,25].

Some limitations to the present study must be noted. First, there is potential for smoking group misclassification due to categorization of smoking status at baseline and 12-month follow-up. The “new smokers” group may contain intermittent smokers should individuals not be smoking for the first time. After adjusting for age, sex, baseline symptoms and health services use, the new smokers had significantly higher odds of wheeze, chest tightness, night-time symptoms, asthma attacks and walk-in clinic visits compared to the non-smokers. Although not included in Table 3, we also conducted comparisons between the new smokers and all baseline smokers using multivariable mixed effect analysis. If these new smokers were indeed intermittent smokers being misclassified as new smokers, one would anticipate the odds ratios to be closer to one. The odds of asthma outcomes while lower were in fact similar to those between new smokers and non-smokers reported in Table 3. While there may be some level of potential misclassification, the observed differences between the groups cannot be completely explained by misclassification.

Since there may be important clinical differences between someone who stopped smoking a long time ago versus someone who stopped recently, it would be helpful to know time of smoking cessation. Collecting smoking history and more specifically, the number of packs of cigarettes smoked by smokers, ex-smokers, new- and intermittent smokers, would be useful. However, collecting smoking history retrospectively may not be ideal as it is prone to recall bias. The number of baseline non-smokers who were former ex-smokers or never-smokers may also influence significant findings; nevertheless, the benefit of adding a ”former” or “never” smokers group is limited by the number of individuals who fit the criteria.

Second, due to the design of the study, there is potential for interviewer bias. To avoid interviewer bias, one may consider blinding the interviewer to the status of the patients (case or control) which then would avoid differential probing. In our study, however, it was impractical to blind the interviewers (nurses and asthma educators) since these professionals recruited asthma patients into the study. The smoking status of the patients was, however, unknown to the interviewers prior to the interview and the information about smoking was collected after all other information was completed. In our study, we used standardized questionnaires to collect information on asthma symptoms, health services use and smoking status. The interviewers were not allowed to deviate from the scripts and thus, we did what was possible in a community-based study.

Finally, additional information about lung function and second-hand smoke was not included in the current study. We did not collect lung function data such as patient spirometry tests, and therefore, could not adjust for asthma severity when evaluating patients’ symptoms and asthma control. However, our findings are consistent with results reported by other studies that demonstrate correlation between the improvement in lung function measured objectively with “feeling better” after smoking cessation [6]. Additionally, although we collected second-hand smoke information in the current study, it was not a variable accounted for in our findings. The absolute number of subjects not exposed to second-hand smoke in the ex-smokers group was 4, whereas it was 2 in the new smokers group. We attempted to include the second-hand smoke variable in our multivariable analysis, however, the small numbers did not allow the model to converge and we were not able to obtain the risk estimate on smoking status. Incorporating these variables as covariates in future larger studies would be an asset. In the current study, there were few individuals who changed smoking status in a 12-month period and thus, there may be a lack of power to detect an effect. A larger study would need to be conducted in order to have adequate power to adjust for the spectrum of asthma-related symptom and exposure variables.

## Conclusions

Our study provides measurable and clinically important findings that associate smoking cessation among asthma patients with improved asthma control. Asthma patients who are going through smoking cessation may require ongoing counselling, support and review of personalized quit plan. The monitoring of smoking cessation programs should be integrated in the regular asthma follow-up care patients receive by health practitioners. Additionally, health care practitioners should closely monitor smoking cessation to monitor and quantify the long-term effectiveness of smoking cessation on asthma control. Furthermore, health practitioners and asthma programs can use powerful education messages to emphasize the benefits of smoking cessation as a priority to current smokers.

## Competing interests

The authors declare that they have no competing interests.

## Authors’ contributions

TT planned the study, carried out the analysis and co-wrote the paper. CD assisted in the interpretation of the results and co-wrote the manuscript. RF and SM assisted with edits in the manuscript. All authors read and approved the final manuscript.
